# Moiré superlattices at the topological insulator Bi_2_Te_3_

**DOI:** 10.1038/srep20278

**Published:** 2016-02-08

**Authors:** Koen Schouteden, Zhe Li, Taishi Chen, Fengqi Song, Bart Partoens, Chris Van Haesendonck, Kyungwha Park

**Affiliations:** 1 Solid-State Physics and Magnetism Section, KU Leuven, BE-3001 Leuven, Belgium; 2 National Laboratory of Solid State Microstructures, Collaborative Innovation Center of Advanced Microstructures, and Department of Physics, Nanjing University, Nanjing 210093, China; 3 Department of Physics, Universiteit Antwerpen, Groenenborgerlaan 171, B-2020 Antwerpen, Belgium; 4 Department of Physics, Virginia Tech, Blacksburg, Virginia 24061, USA

## Abstract

We report on the observation of complex superlattices at the surface of the topological insulator Bi_2_Te_3_. Scanning tunneling microscopy reveals the existence of two different periodic structures in addition to the Bi_2_Te_3_ atomic lattice, which is found to strongly affect the local electronic structure. These three different periodicities are interpreted to result from a single small in-plane rotation of the topmost quintuple layer only. Density functional theory calculations support the observed increase in the DOS near the Fermi level, and exclude the possibility that strain is at the origin of the observed Moiré pattern. Exploration of Moiré superlattices formed by the quintuple layers of topological insulators holds great potential for further tuning of the properties of topological insulators.

The exotic electronic properties of topological insulators (TIs)^1^ hold great promise for future technological developments and consequently they have been a topic of increasing scientific interest in the past years. Similarly to the much investigated graphene, TIs have a layered structure with relatively weak interlayer coupling, implying that the layers can be easily exfoliated using, e.g., adhesive tape^2^. It has already been shown that the electronic properties of (multi-layer) graphene are strongly influenced by its stacking on the supporting substrate^3^ as well as by the precise stacking of the graphene atomic layers on top of each other^4^. In both cases a periodic Moiré-type superlattice emerges as a result of the large-scale commensurability of the stacked atomic lattices. This leads to a periodically varying degree of hybridization between (the atoms in) the stacked layers or even to periodic strain-induced Moiré “blistering” as is the case for graphene on Ru^5^. As a result, a (lateral) superlattice emerges with a periodicity that is larger than that of the two lattices involved. The periodic potential related to the superlattice can induce new electronic properties in the constituting material, e.g., Van Hove singularities^4^,^6^, band gaps^7^, and minibands^3^.

While Moiré superlattices and their impact on the electronic properties have already been investigated in great detail for a variety of materials, only very limited studies exist for superlattices formed in topological materials. Thus far, Moiré patterns have been mostly reported for thin Bi_2_Se_3_ films on various substrates including graphene^8^, FeSe^9^, Au(111)^10^, h-BN^11^ and NbSe_2_^9^,^12^, caused by the direct lattice mismatch between the Bi_2_Se_3_ and the substrate. It was predicted that a Moiré superlattice can lead to topologically nontrivial subbands in transition-metal dichalcogenides^13^. Very recently, it was shown that dislocation-related strain in Bi_2_Se_3_ films grown on SiC(0001)^14^ is accompanied by the emergence of a Moiré-type superlattice and may allow tuning of the Dirac states of the TI. The strain may also lead to the emergence of a pseudo-magnetic field and the appearance of Landau levels as demonstrated for the case of graphene^5^,^6^,^15^,^16^,^17^. To the best of our knowledge, to date only one example of a Moiré pattern has been reported for the case of a bulk-type (thick film) topological insulator sample (see Fig. 1a in ref. 14), which indicates that Moiré patterns are less likely to occur spontaneously for topological insulators compared to (multi-layer) graphene.

Here we demonstrate the existence of a complex Moiré-type superlattice for the bulk single crystal TI Bi_2_Te_3_ relying on scanning tunneling microscopy (STM) and spectroscopy (STS). The superlattice can be related to an in-plane rotation of the topmost quintuple layer (QL) of the TI, which is found to strongly modify its electronic structure. Our experimental findings are corroborated by density functional theory (DFT) calculations.

## Results and Discussion

**Figure 1:**
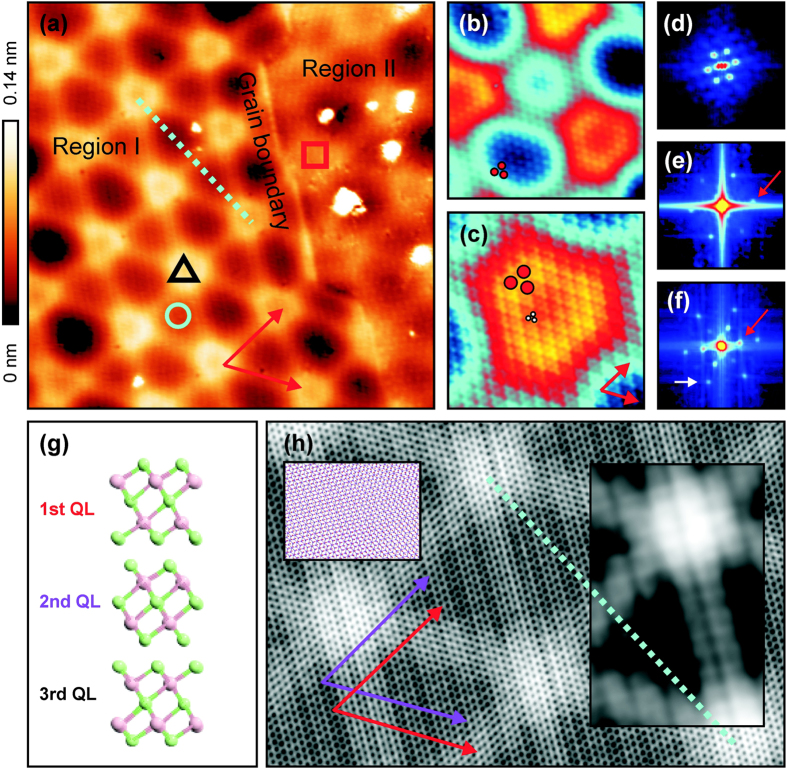
(**a**–**c**) STM topography images of a Moiré superlattice at the Bi_2_Te_3_ surface. Image sizes are 60 × 60 nm^2^, 20 × 20 nm^2^ and 10 × 10 nm^2^, respectively. Tunneling setpoints are 1.1 V and 0.13 nA, 1.1 V and 0.5 nA, and 0.1 mV and 0.2 nA, respectively. (**d**–**f**) Corresponding Fourier-transform images. Image sizes are 0.7 × 0.7 nm^−2^, 5.3 × 5.3 nm^−2^ and 9.1 × 9.1 nm^−2^, respectively. (**g**) Schematic model structure of three Bi_2_Te_3_ QLs (viewpoint: parallel to the QLs). Green (pink) spheres represent Te (Bi) atoms. (**h**) Simulated Moiré pattern with an in-plane rotation of 1.2°. A smoothing filter was applied to better resolve the Moiré patterns. Left inset: Close-up view of the simulated Moiré pattern prior to smoothing. Red (purple) circles belong to the first (second) set of three atomic layers. Right inset: Simulated pattern after applying a more heavy smoothing filter.

Figure 1(a) presents a large-scale STM topography image of the Bi_2_Te_3_ surface. The few nanosized particles that are discerned in the image can be associated with Cu dopant atoms that were introduced in the Bi_2_Te_3_ sample during the crystal growth and that were previously shown to form Cu clusters in between the QLs during ageing of the sample^18^. This is accompanied by a suppression of the bulk conductance of the material by up to four orders of magnitude. The atomic structure of three Bi_2_Te_3_ QLs is schematically presented in Fig. 1(g). Interestingly, a pronounced periodic structure can be clearly resolved in Fig. 1(a), together with an atomically sharp boundary that separates two regions with different appearance of the superstructure [labeled as Region I and Region II in Fig. 1(a)]. Bright (truncated) triangular shaped features in region I are organized in a triangular lattice and are interconnected by bright lines. The observed boundary may be attributed to the grain boundary between two different crystalline domains that are known to exist in Bi_2_Te_3_ flakes^18^. This boundary differs from previously reported grain boundaries in Bi_2_Se_3_ (0001) films that consist of arrays of alternating edge dislocation pairs. Those dislocations were found to locally introduce periodic in-plane compressive and tensile strains, giving rise to a simpler Moiré pattern^14^. In addition, our observed Moiré-type pattern appears more complex than previously reported patterns formed by a thin Bi_2_Se_3_ film on a graphene^8^ or FeSe^9^ substrate.

The Cu dopants in our Bi_2_Te_3_ sample form clusters randomly located in between the QLs^18^, and so they are not directly related to the here observed periodic superstructures [large-scale STM topographies of regions with and without a Moiré pattern are presented in Fig. S2 in the Supplementary Information (SI)]. However, the intercalated Cu particles may reduce an interlayer coupling and thus affect the probability for in-plane rotation of the topmost QL. In our series of STM experiments we could retrieve only exceptionally a Moiré superlattice on our samples, i.e., near a grain boundary in the Bi_2_Te_3_ surface [Fig. S3 in the SI presents an STM topography image of another Moiré region that is 400 nm away from the region shown in Fig. 1]. Such grain boundaries appear rarely on our samples and are difficult to retrieve with STM. The formation of a Moiré superlattice at the Bi_2_Te_3_ surface can likely be promoted by selectively tuning the preparation conditions of the Bi_2_Te_3_ crystal, e.g., by further intercalating the QLs of the TI^19^. This is required for more systematic investigation of Moiré superlattices at TI crystals and to obtain a detailed understanding of their impact on the electronic structure. Nevertheless, our findings illustrate the feasibility and potential importance of Moiré superlattices formed by QLs of TIs, as will be discussed in detail in the following.

The periodicity of the large superstructure in Fig. 1(a) can be identified by the appearance of six bright spots in the corresponding Fourier-transform image in (d). The mean periodicity of the bright (truncated) triangular shaped features is 13 ± 1 nm. Upon more careful inspection of Fig. 1(a), an additional, less pronounced structure with a smaller periodicity can be discerned in Region I and in Region II. This less pronounced superstructure can be better resolved in Fig. 1(b), which is a close-up view of Region I, and is highlighted by three solid red circles. From the corresponding Fourier-transform image shown in (e) (one of the six related Fourier maxima is highlighted by the red arrow), a periodicity of 0.8 ± 0.1 nm can be inferred (compared to about 1.3 ± 0.1 nm in Region II). The atomic structure of the Bi_2_Te_3_ surface can be resolved upon further zooming in on the same area with higher resolution, see Fig. 1(c). Red arrows in (a) and (c) indicate two directions of the atomic lattice. The atomic structure in (c) is highlighted by three solid white circles. The corresponding Fourier-transform image in (f) reveals two sets of bright dots. The inner set (one of the six related Fourier maxima is highlighted by the red arrow) stems from the superstructure periodicity of 0.8 nm, while the outer set (one of the six related Fourier maxima is highlighted by the white arrow) arises from the atomic lattice periodicity. An atomic periodicity of about 0.4 nm can be derived, consistent with previous STM observations of the Bi_2_Te_3_ surface^20^. Note that in STM images of pristine Bi_2_Te_3_ surfaces without a Moiré superstructure only either tellurium or bismuth atoms are resolved as protrusions, depending on the sign of the applied tunneling voltage^20^. The appearance of the Moiré pattern in STM topography images did not reveal a particular dependence on the applied sample voltage in the investigated voltage range spanning from 0.1 V to 1.1 V.

As indicated above, a Moiré superstructure is typically formed whenever two different atomic lattices are stacked on top of each other and when they exhibit a large-scale commensurability. Alternatively, a Moiré pattern can emerge due to a local in-plane distortion of the atomic lattices, e.g., due to folding of multi-layer graphene sheets into a multi-walled carbon nanotube^21^. The here observed superstructure manifests itself over relatively large distances across the flat TI surface, which in this case is indicative of an in-plane rotation of the top layers with respect to each other^4^.

Three different periodicities can typically be achieved by two successive in-plane rotations of both top stacked layers, as is the case for, e.g., stacked graphene layers^22^. Here we find that three different periodicities can result from a small in-plane rotation of only a single topmost QL, if it consists of ABC stacked atomic layers. Therefore we constructed a Moiré superstructure by rotating a set of three atomic layers that are ABC stacked by a small in-plane rotation angle with respect to another set of three atomic layers with ABC stacking. Considering that each set of three atomic layers with ABC stacking corresponds to one QL, our simulation mimics an in-plane rotation of the topmost QL relative to the next QL. We find that an in-plane rotation angle of 1.2° provides a Moiré pattern with a periodicity of 12.5 nm, which agrees very well with the experimental periodicity of 13 ± 1 nm. The result is presented in Fig. 1(h). A smoothing operation was applied to optimize the visualization of the simulated image (a small non-smoothed region of the simulated image is shown in the left inset). Red (purple) arrows indicate the atomic directions of the first (second) set of three atomic layers. Red arrows in (h) have the same orientation as in (a) and (c). Clearly a triangular lattice of bright spots that are connected by bright lines can be resolved, in good agreement with the experimental observation. The blue dotted line in (a) and (h) has the same orientation and indicates the Moiré pattern with large periodicity. The black dots in (h) represent the surface atoms that have a periodicity of about 0.44 nm. Interestingly, we also uncover a smaller periodicity of about 0.8 nm in Fig. 1(h) (running parallel to the large bright lines), which is in good agreement with the 0.8 nm periodicity of the red dots in Fig. 1(c). The pattern with smaller periodicity is better resolved in the right inset in (h), which was subject to a heavier smoothing operation. We note that the QL structure with ABC stacking seems to be essential for the observed Moiré pattern because an in-plane rotation of two atomic layers alone can produce neither the Moiré pattern with the two different periodicities, nor reproduce the bright lines connecting the bright spots in (a) and (h). Furthermore, our simulation shows that two successive in-plane rotations of top two ABC stacked layers relative to unrotated ABC stacked layers give rise to a Moiré pattern more complex than the experimentally observed pattern (data not shown). Finally, small discrepancies between the experiment and the simulation [the bright spots in (h) do not have the truncated triangular shape observed in (a)] can be related to the simplicity of the approach. Nevertheless a good agreement is achieved between experiment and simulation, which allows us to conclude that the observed patterns stem from an in-plane rotation of two QLs with respect to each other. A simulation approach that goes beyond our present approach and that allows to relax the atomic model may allow to achieve even better agreement between simulation and experiment. Local reconstruction of the surface atoms may even lead to the emergence of chiral effects in the formed Moiré patterns^23^, yet such effects are not resolved within our accuracy.

**Figure 2:**
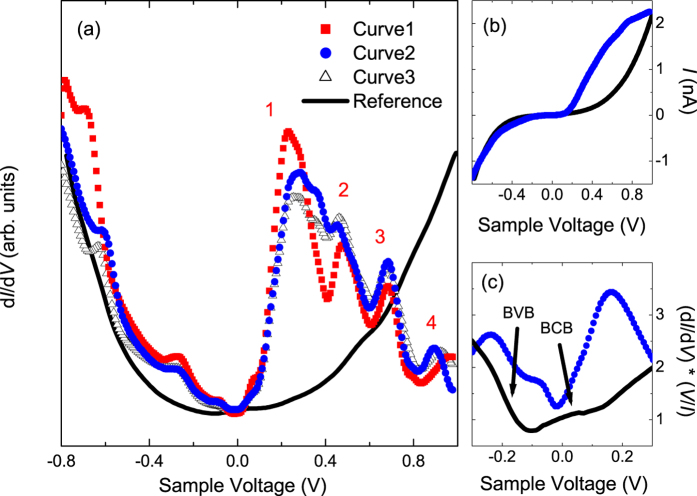
(**a**) d*I*/d*V* spectra recorded on the locations indicated in Fig. 1(a), as well as a reference spectrum recorded on an area without Moiré pattern on the same sample. Tunneling setpoints are 1.0 V and 0.75 nA (0.25 nA for the reference spectrum). (**b**,**c**) Corresponding *I*(*V*) spectra and normalized (d*I*/d*V*)(*I*/*V*) spectra, respectively. The reference curves in (**a**) and (**b**) are set are rescaled vertically to allow for easy comparison with the spectra recorded on the Moiré patterned regions.

To elucidate the effect of the Moiré superstructures on the electronic properties of the Bi_2_Te_3_ surface, we relied on STS measurements that probe the local density of states (LDOS) of the sample surface. Figure 2(a) presents three d*I*/d*V* spectra that each are an average of 10 single spectra that are recorded in a broad voltage range in the regions indicated in Fig. 1(a) (a smaller-voltage range version of such a spectrum was already presented in Fig. 3(c) in ref. 18), as well as a reference spectrum that is recorded on a region that does not exhibit a Moiré pattern. Clearly, there exist striking differences in the d*I*/d*V* spectra: While the reference spectrum shows a more or less parabolic voltage-dependence, the Moiré-region spectra exhibit several very pronounced electronic resonances throughout the entire investigated voltage range. The energy positions of the resonances show only minor variation across the Moiré superlattice in a particular region, while differences between Region I and Region II are more noticeable (see Fig. 2(a)).

Figure 2(b) shows that the corresponding *I*-*V* spectra follow a pronounced non-linear behavior. This implies that the relative heights of the electronic features in the derived (d*I*/d*V*)(*V*) spectra (often used as a direct measure of the LDOS of the sample) depend on the setpoint used for the sample voltage (e.g., positive or negative voltage setpoint). Therefore, for more detailed comparison of the spectral features we need to account for this setpoint-dependence, which can be done by relying on normalized (d*I*/d*V*)*(*V*/*I*) spectra that yield a better measure of the LDOS^24^. Figure 2(c) presents a normalized spectrum of the Moiré region and a reference spectrum of a region without Moiré pattern. The reference spectrum has a local minimum around −0.1 V, in agreement with previously reported spectra for Bi_2_Te_3_^20^. The small rise in the slope near +0.1 V can be related to the bottom of the bulk conduction band (BCB), while the top of the bulk valence band (BVB) at the Γ-point that is expected around −0.2 V^20^ appears hidden in the steeply rising slope [the BVB and BCB are indicated by arrows in Fig. 2(c)]. As already indicated above, the experimental data presented in this work were obtained on a Bi_2_Te_3_ sample that was doped with Cu, i.e., Sample 4 in Table 1 of ref. 18. Considering that neutral Cu dopant particles are located within the van der Waals gap between the QLs^18^ and considering the similarity of our (d*I*/d*V*)(*V*) data with previously reported data for undoped Bi_2_Te_3_^20^, it seems unlikely that the Cu dopants significantly shift the Dirac point. The Dirac point is therefore expected to be located in between the top of the BVB at the Γ point and the bottom of the BCB.

The appearance of pronounced electronic resonances in the Moiré-patterned regions shows resemblance to the pronounced resonances previously observed on graphene nanobubbles^17^. Such bubbles can be formed starting from a periodic Moiré-blistered graphene layer on a Ru substrate^5^. By intercalation with oxygen the Moiré blisters further evolve into larger nanobubbles, giving rise to significant strain in the bubbles. The origin of the series of peaks has been associated with Landau levels that arise from the strain-induced pseudo-magnetic field present in these systems^13^,^15^,^16^. The here observed Moiré patterns might be associated with local strain in the Bi_2_Te_3_ TI. The positions of the pronounced peaks of curve 1 above the Fermi level at zero voltage [labeled *n* = 1 to 4 in Fig. 2(a)] follow a linear behavior as a function of their Landau level index number *n*^25^ [based on the present set of data we cannot discriminate whether the peaks follow a linear behavior with *n*, 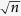, 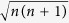 or 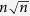 (fitting results are not shown)]. However, compared to the graphene case, the here observed Moiré patterns reveal very limited height variations in the STM topography images (around 0.1 nm compared to ≥0.4 nm for graphene^5^,^17^) and so large strain effects are not expected for the present system. As a result, interpretation of the here observed peaks in terms of Landau levels seems unlikely. This is also supported by our DFT calculations.

**Figure 3:**
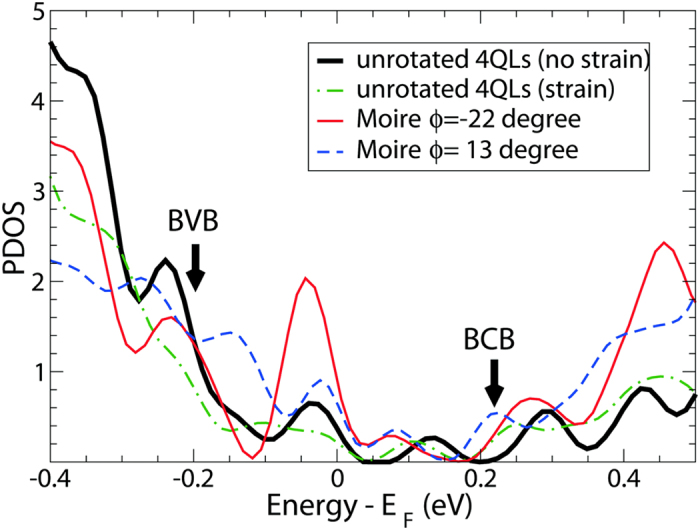
Projected density of states (PDOS) per 16.66 Å^2^ for Bi_2_Te_3_ systems consisting of 4 QLs, of which the topmost QL was rotated in-plane either by −22° (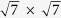 supercell) or by +13° (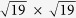 supercell) in comparison to PDOS for the unrotated 4-QL slabs without strain and with an in-plane strain of 2%. The PDOS is onto the top 2 QLs. The energy is relative to the Fermi level *E*_*F*_, while the unit of the PDOS is arbitrary. The top of the BVB at Γ and the bottom of the BCB for the unrotated 4-QL without strain are indicated with arrows.

To interpret the experimental STM and STS observations we performed DFT calculations of the Bi_2_Te_3_ (0001) surface. We considered 4 QLs, of which the lower 3 QLs are kept fixed and the topmost QL is rotated by an in-plane rotation angle. Two rotation angles were considered, i.e., −22° and +13°, for which 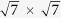 and 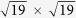 supercells were used, respectively. Here we note that it is computationally not feasible to calculate the 1.2° rotation because of the very large supercell size, i.e., much larger than that for −22**°** and +13**°** rotations. The calculated projected density of states (PDOS) onto top 2 QLs of both systems are presented in Fig. 3. We checked that the projected density of states for the 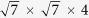-QLs supercell is very similar to that for the 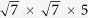-QLs supercell when only the topmost QL is rotated in both cases. For comparison the corresponding PDOS was also computed for an unrotated 4-QL slab without strain (black solid curve) and with an in-plane compressive strain of 2% (green dash-dotted curve). Due to the small thickness of the 4-QL slab, the bottom of the BCB appears at a higher energy than in the experimental data. With both rotation angles an increase of DOS is observed around the Fermi level, despite some dependence of the features on the rotation angle. The increased DOS near the Fermi level is in agreement with the experimentally observed increase of the DOS near zero voltage for the Moiré-patterned regions [see Fig. 2(c)].

**Figure 4:**
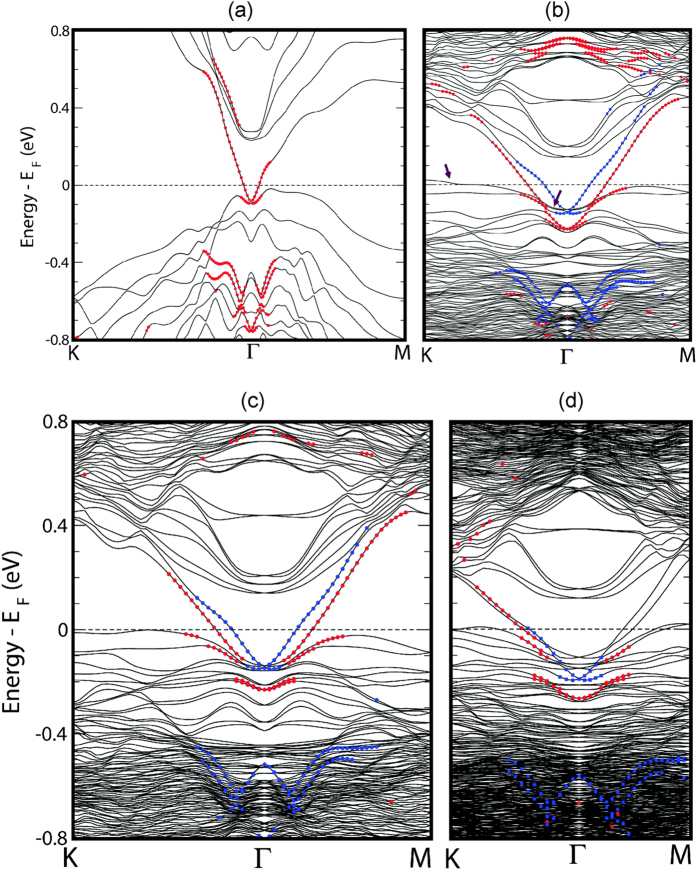
(**a**) DFT-calculated band structure of a pristine non-rotated slab of 4 QLs, where the red symbols represent the surface states localized onto either the topmost or the bottommost QL. The bands are doubly degenerate due to the time-reversal and inversion symmetry. (**b**) DFT-calculated band structure of a slab of 4 QLs with an in-plane rotation angle of 22 degrees and 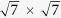 surface atoms per unit cell. (**c**) DFT-calculated band structure of 5 QLs with the same in-plane rotation angle as in (**b**). (**d**) DFT-calculated band structure of a slab of 4 QLs with an in-plane rotation angle of 13 degrees and 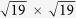 surface atoms per unit cell. In (**b**–**d**), only the topmost QL was rotated, and the red (blue) symbols indicate the surface states localized onto the topmost (bottommost) QL. The bands in (**b**–**d**) are not double degenerate due to the broken inversion symmetry caused by the in-plane rotations.

The sudden increase of the DOS just below and above the Fermi level in Fig. 2(c) may originate from quantum-well states that emerge near the Fermi level in the vicinity of the zone boundaries such as K and M as well as near Γ. The results of band structure calculations are presented in Fig. 4 below. We computed the band structures of the two 4 QLs with different rotation angles, 22 degrees and 13 degrees, and of the 5 QLs with a rotation angle of 22 degrees, and compared them with that of a pristine un-rotated 4 QLs of Bi_2_Te_3_. Note that the bands in the non-rotated slab have double degeneracy due to time-reversal and inversion symmetries, whereas the bands with in-plane rotations of the topmost QL are not degenerate due to broken inversion symmetry. In contrast to the band structure of the non-rotated slab, the rotated slab band structures show that a pair of quantum-well states highly localized onto the top two QLs (topmost and topmost-1) marked by the arrows in Fig. 4(b), appear near the Fermi level in the vicinity of the zone boundaries such as K and M, as well as of the Γ point, and that those quantum-well states are also flattened along the K-Γ and Γ-M lines. In addition, for the rotated slabs, the Dirac cones as well as the quantum-well states in the bulk conduction and valence region are shifted downward relative to the Fermi level when compared to those for the non-rotated slab. From our calculated band structure, we infer that there exists a potential difference between the topmost QL (rotated) and the bottommost QL caused by the in-plane rotations. As a result, the Dirac cones as well as the bulk-like quantum-well states in the bulk conduction and valence band regions are shifted downward relative to the Fermi level, compared to those for the unrotated slab. This feature is observed for all three supercells that we considered: 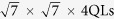, 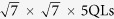, and 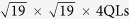 (Fig. 4(b–d)). The several quantum well states close to the Fermi level are likely responsible for the sudden increase of the density of states right below and above the Fermi level shown in Figs 2(c) and 3 above.

Finally, we find that the PDOS of the rotated 4-QL structures significantly differs from that of the strained unrotated slab. As illustrated in Fig. 3, the PDOS of the strained slab is similar to that of the unstrained slab, yet the height of the spectral features is reduced, which is in contrast to the experimental observations. Therefore, we conclude that the PDOS of the rotated structures reflect the important features of the sample PDOS near the Fermi level, while the sample PDOS appears far less sensitive to strain effects. Given the complexity of the system, a full correspondence between theory (PDOS) and experiment (STS spectra) is not feasible at this stage. Nevertheless, the calculations support interpretation of the experimentally observed Moiré superlattice in terms of an in-plane rotation of the topmost Bi_2_Te_3_ QL.

In summary, by using STM and STS we have shown that complex Moiré superlattices can exist at the surface of the TI Bi_2_Te_3_. The superlattices give rise to a strongly modified electronic structure. DFT calculations support the interpretation of the experiments that an in-plane rotation of the topmost QL induces the Moiré-type superlattice and the accordingly modified electronic structure.

## Methods

### Sample Preparation

The Bi_2_Te_3_ crystal was prepared by the melting method as described in detail in ref. 18 (Sample 4 in Table 1). In brief, mixed Bi, Te and Cu powders (99.999% purity, Alfa Aesar, with a molar ratio of 2:3:0.15) were sealed in a silica ampoule. The material was heated to 850 °C for 3 days while being stirred at a speed of 5 turns per minute. After cooling to 550 °C in 9 days, the material was kept at 550 °C for 5 days. After ageing the flakes in vacuum at room temperature for about 600 days, the flakes were exposed to air. To obtain a clean sample surface, the flakes were exfoliated *ex-situ* with adhesive tape, after which they were mounted within a few minutes in the ultra-high vacuum (UHV) STM setup. To desorb possible surface contaminants due to the short exposure of the freshly cleaved sample to ambient conditions, samples were annealed to about 150–200 °C for several hours in UHV prior STM measurements. We also performed experiments on undoped Bi_2_Te_3_ that was prepared following the same approach. For the undoped sample we find very flat and atomically clean surfaces that are ideally suited for cryogenic STM experiments (STM topographies of undoped Bi_2_Te_3_ are presented in Fig. S1 in the SI). We note, however, that the presence of a limited degree of surface contamination in Fig. 1(a) cannot be excluded, yet this does not affect our experimental observation and interpretation of the Moiré superstructures.

### Sample Characterization

All experiments were conducted in a UHV system (base pressure in the 10^−11^ mbar range) that includes a low-temperature STM (Omicron Nanotechnology) operated at 4.5 K. *I*(*V*) spectra were recorded with open feedback loop, from which normalized (d*I*/d*V*)/(*I*/*V*) spectra that reflect the LDOS can be obtained^24^. In addition, (d*I*/d*V*)(*V*) spectra were acquired by lock-in detection with an open feedback loop (amplitude is typically about 40 mV) at 800 Hz. All STM/STS data in this work were obtained with polycrystalline W tips that were electrochemically etched and cleaned *in-situ* by thermal treatment. All bias voltages mentioned are with respect to the sample, and the STM tip is virtually grounded. The STM images were analyzed using the Nanotec WSxM software^26^. In total, a surface area of about 510^5^ nm^2^ (typical scan size is 100 × 100 nm^2^) was investigated (including about 10 tip approaches on different locations of the sample), of which only an area spanning about 510^3^ nm^2^ (comprising two regions measured within one tip approach) revealed the here reported Moiré patterns.

### Calculations

DFT calculations on Bi_2_Te_3_ were carried out by using the DFT code *VASP*^27^,^28^, within the generalized gradient approximation (GGA)^29^ for an exchange-correlation functional and with projector-augmented wave (PAW) pseudopotentials^30^. Spin-orbit coupling was considered self-consistently in the DFT calculations. The lattice constants of Bi_2_Te_3_ slabs were taken from experimental data^31^. All the slabs considered have a thickness of 4 QLs, i.e., about 4 nm, unless specified otherwise. For a 4-QL slab (unstrained or in-plane strained) with a 1 × 1 surface atom, an energy cutoff of 300 eV and 15 × 15 × 1 *k*-points sampling (based on Monkhorst-Pack scheme) were used with a vacuum layer of 30 Å. To simulate the effect of strain, a compressive strain of 2% was applied to the 1 × 1 × 4 QL slab along the 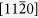 direction with a Poisson ratio of 0.25. The geometry of the in-plane strained structure was not relaxed. For 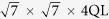, 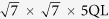, and 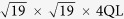 supercells, a vacuum layer 2.0–2.2 nm thick was included. An energy cutoff of 175.0 eV was used, and 3 × 3 × 1 *k*-points including the Γ point were sampled for both supercells. The total energy converges down to 10^−5^ eV.

## Additional Information

**How to cite this article**: Schouteden, K. *et al.* Moiré superlattices at the topological insulator Bi_2_Te_3_. *Sci. Rep.* **6**, 20278; doi: 10.1038/srep20278 (2016).

## Supplementary Material

Supplementary Information
