# Supplementary material for: Moiré superlattices at the topological insulator Bi2Te3
Source: Sci Rep. 2016 Feb 8;6:20278. doi: 10.1038/srep20278 (PMC4745016; doi:10.1038/srep20278)
Supplement: Supplementary Information [file srep20278-s1.pdf]

Supplementary Information for manuscript
"Moiré superlattices at the topological insulator Bi_2Te_3"

Koen Schouteden[1,*], Zhe Li[1], Taishi Chen[2], Fengqi Song[2], Bart Partoens[3], Chris Van Haesendonck[1], and Kyungwha Park[4]

[1] Solid-State Physics and Magnetism Section, KU Leuven, BE-3001 Leuven, Belgium

[2] National Laboratory of Solid State Microstructures, Collaborative Innovation Center of Advanced Microstructures, and Department of Physics, Nanjing University, Nanjing 210093, China

[3] Department of Physics, Universiteit Antwerpen, Groenenborgerlaan 171, B-2020 Antwerpen, Belgium

[4] Department of Physics, Virginia Tech, Blacksburg, Virginia 24061, USA

[*] Koen.Schouteden@fys.kuleuven.be

Table of Contents:

S1. STM topographies of undoped Bi_2Te_3

S2. Large-scale STM topographies of regions with and without Moiré pattern

S3. STM topography of another Moiré region

S1. STM topographies of undoped Bi_2Te_3

In our STM setup in-situ cleaving is not possible. Therefore, to desorb possible surface contaminants due to the short exposure of the freshly cleaved sample to ambient conditions, we have applied a mild in-situ annealing. To verify the cleanliness of thus obtained Bi_2Te_3 surfaces, we have performed experiments on an undoped Bi_2Te_3 sample that was prepared following this approach. For the undoped sample we find very flat and atomically clean surfaces that are ideally suited for cryogenic STM experiments. This is illustrated in Figure S1 below. We want to note here that we never observed a Moiré pattern in our experiments with the undoped Bi_2Te_3 surface.

For the Cu-doped samples we found many atomic-size particles on the surface, which were previously attributed to small Cu aggregates that were formed by the Cu dopants in between the quintuple layers during ageing of the sample (this was explained in detail in Ref. [18] in the manuscript). It is therefore likely that the particles observed on the Moiré-regions of the sample (Fig. 1a in the manuscript) are Cu aggregates as well. However, the presence of a limited degree of surface contamination cannot be excluded. Nevertheless, this does not affect our experimental observation and interpretation of the Moiré superstructures.

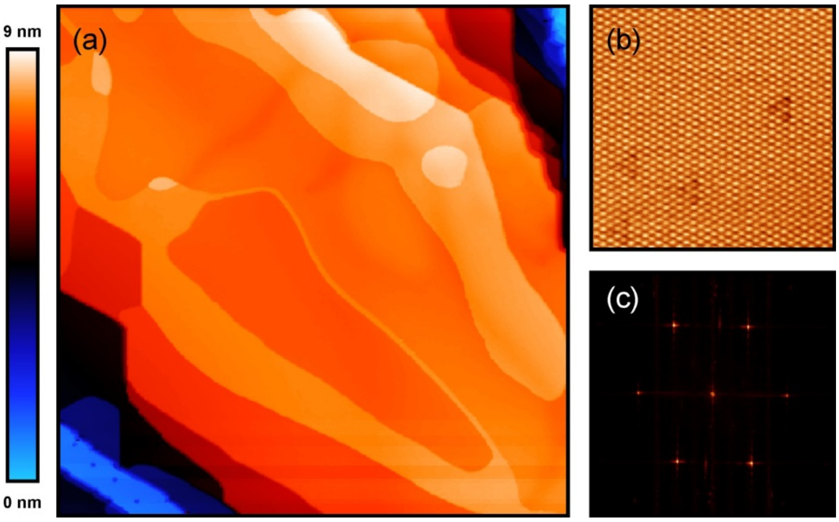

Figure S1. (a) 500 x 500 nm^2 STM topography image of undoped Bi_2Te_3. The sample was cleaved ex situ with adhesive tape. Mild annealing in UHV was applied prior to STM experiments. (b) 14 x 14 nm^2 STM atomically resolved close-up view of the clean Bi_2Te_3 surface. (c) Fourier-transform image of (b). Inferred atomic periodicity is 0.42 +/- 0.01 nm, in agreement with previous reports (e.g., Ref. [19] in our manuscript).

S2. Large-scale STM topographies of regions with and without Moiré pattern

Figures S2 (a) and (b) present a large-scale STM topography of a Bi_2Te_3 region without and with a Moiré pattern, respectively. We did not achieve large-scale atomic resolution imaging of the Moiré-free regions of the same sample, since this was hampered by the relatively high density of Cu aggregates in these regions. Nevertheless, within the obtained resolution it is clear that a large-periodicity Moiré pattern is lacking in the left figure (both images have the same color height scale), which is a typical STM image of the investigated sample that is discussed in the manuscript. We note that the crystalline quality of the undoped and Cu-doped Bi_2Te_3 samples have been checked in detail in a previous work (Ref.[18] in the manuscript) and it was demonstrated that the Cu-dopants do not alter the crystalline structure of the Bi_2Te_3. **In our future research we aim - on new samples - to achieve large-scale atomic resolution images of both reconstructed Moiré regions and unreconstructed Moiré-free regions.**

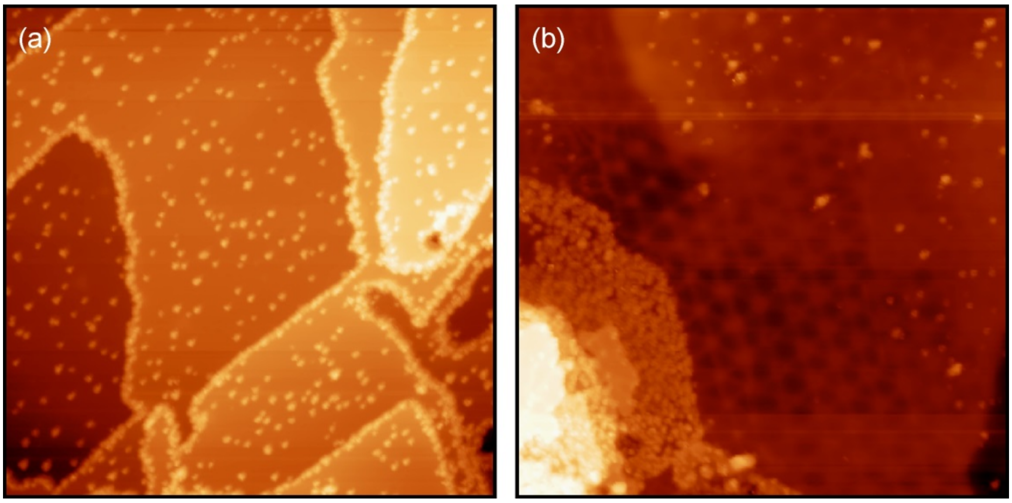

Figure S2. (a) and (b) 200 x 200 nm^2 STM topography images of regions of the same sample, without and with a Moiré pattern, respectively. Color height scale spans 2.2 nm for both images.

S3. STM topography of another Moiré region

Figure S3 presents an STM topography image of a region that is 400 nm away from the region shown in Fig. 1 of the manuscript and Fig. S2 (b) above. Although the resolution of the image is less good, a periodic superstructure can be clearly resolved.

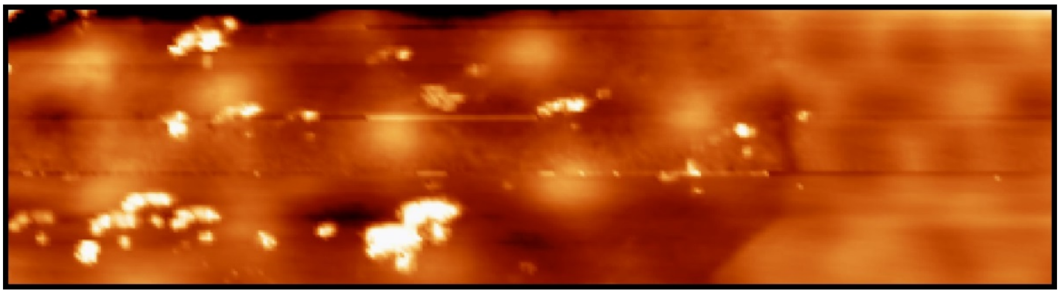

Figure S3. 100 x 25 nm^2 STM topography image of the surface of Cu-doped Bi_2Te_3 for a region that is 400 nm away from the region shown in Fig. S2 (b) above.
